# Neurodevelopmental outcome at 2 years of age in preterm infants with late-onset sepsis

**DOI:** 10.1007/s00431-019-03339-2

**Published:** 2019-02-18

**Authors:** I. A. Zonnenberg, E. M. van Dijk-Lokkart, F. A. M. van den Dungen, R. J. Vermeulen, M. M. van Weissenbruch

**Affiliations:** 1 Department of Neonatology, Emma Children’s Hospital, Amsterdam UMC, Vrije Universiteit Amsterdam, De Boelelaan 1117, 1081 HV Amsterdam, The Netherlands; 2 Department of Medical Psychology, Amsterdam UMC, Vrije Universiteit Amsterdam, Amsterdam, The Netherlands; 3 Department of Child Neurology, Neuroscience Campus Amsterdam, Emma Children’s Hospital, Amsterdam UMC, Vrije Universiteit Amsterdam, Amsterdam, The Netherlands; 4 Present Address: Child Neurology, Department of Neurology, MUMC+, Maastricht, The Netherlands

**Keywords:** Late-onset sepsis, Neurodevelopmental outcome, Preterm infant

## Abstract

Late-onset sepsis is associated with impaired neurodevelopmental outcome in preterm infants. This prospective cohort study aims to establish the effect of sepsis after 72 h of life on cognitive, psychomotor, and language development of preterm infants (below 32 weeks gestational age and/or below 1500 g). At 2 years corrected age, neurodevelopmental outcome was tested using Bayley’s Scales of Infant Development-II, Lexilijst (lexical development questionnaire), and behavior checklists. Of 117 patients included, 85 experienced blood culture–proven infection. Coagulase-negative staphylococci were responsible for 55% of the episodes. No significant differences were found in cognitive, motor, and behavioral scores or lexiquotient comparing patients with versus no proven infection. When comparing three groups (coagulase-negative staphylococci, other, and negative blood culture), a significant difference was found in composite cognitive scores (*p* = 0.016), in favor of the coagulase-negative staphylococci group versus other causal agent group (*p* = 0.007). No significant differences were found in other subscales.

*Conclusion*: In this cohort, no differences were found in neurodevelopmental outcome at 2 years corrected age between proven and no proven infection groups; confirmation in larger cohorts with a control group is needed. Patients encountering coagulase-negative staphylococci sepsis showed a significant better cognitive outcome compared to other causal agents.

**Table:** 

**What is Known:**	
*• Late-onset sepsis is associated with impaired neurodevelopmental outcome in preterm infants.*	
**What is New:**	
*• Preterm infants encountering late-onset sepsis by coagulase-negative staphylococci show a better cognitive outcome in comparison to other causal infectious agents in this cohort.*	
*• No differences were found in neurodevelopment at 2 years of age in preterm infants with suspected lateonset sepsis, between proven and no proven infection groups. Confirmation is needed in larger cohorts with a substantial control group.*

## Introduction

Long-term neurodevelopmental outcome is one of the most important outcome parameters of neonatal intensive care. The long-term developmental outcome is, however, at risk due to the hazardous events that will occur during admission to the neonatal intensive care unit (NICU). These events are potentially dangerous for the brain development of the infants and may inflict delay in mental and/or psychomotor development. Examples of these events are respiratory or circulatory insufficiency, intracranial hemorrhage, or resuscitation.

Another associated risk factor for impaired neurodevelopmental outcome is sepsis [1, 2]. Preterm and low birth weight infants have a high risk of acquiring late-onset sepsis during their admission to the NICU. The risk of late-onset sepsis is ranging from 33% in preterm infants with a gestational age less than 28 weeks to 60% in preterm infants less than 25 weeks [3]. Late-onset sepsis is also associated with prolonged hospital stay, length of invasive ventilation, and need for invasive devices and parenteral nutrition [3].

The adverse effects of late-onset sepsis might be due to the inflammatory response and effects of this response on the vulnerable developing brain. Damman et al. described an association of intraventricular hemorrhage or periventricular white matter lesions and pro-inflammatory cytokines like IL-6 due to intrauterine infection [4]. A similar mechanism might occur during postnatal infections. White matter injury has a negative impact on cerebral networking and control, and modulation of the motor system and thus on neurodevelopmental outcome [5]. Mitha et al. reported a higher incidence of cerebral palsy (CP) following neonatal sepsis compared to infants without an infectious episode during admittance [6].

In early postnatal period, a tremendous change in brain structure and function is demonstrated [7], which makes the brain vulnerable. Studies showed reduced brain volumes in infants after experiencing sepsis [8]. An important factor accounting for favorable long-term outcome in preterm infants is adequate weight gain and growth of head circumference. Full enteral nutrition provides better weight gain and less postnatal growth restriction [9]. Infection may have impact on adequate weight gain in several ways. Due to infection, enteral nutrition might be less tolerated, leading to cumulative protein and energy deficits, with impaired weight gain. Also, as a consequence, due to longer parenteral nutrition supplementation and the need for central venous lines, the risk to experience a blood stream infection increases [10].

The aim of this study is to analyze whether neurodevelopmental outcome at 2 years corrected age is compromised in preterm infants with suspected late-onset sepsis, comparing infants with proven infection with infants without a proven infection, in particular if certain species of causal microorganism influence long-term outcome.

## Methods

A prospective cohort study was performed collecting data from preterm infants born with a gestational age < 32 weeks and/or < 1500 g admitted to the level III Neonatal Intensive Care Unit of the VU Medical Center between March 2008 and December 2014 suspected of late-onset sepsis. Preterm infants with syndromal or chromosomal abnormalities and congenital metabolic disorders were excluded. The medical ethical committee of the VU University Medical Center approved the study (protocol number 2008/77), and written informed parental consent was obtained during the first days of admittance at the NICU. Patients were included in the study when a suspicion of late-onset sepsis occurred.

Late-onset sepsis was suspected when one of the following clinical symptoms occurred: hypothermia (< 36.5 °C) or hyperthermia (> 37.5 °C), hypotension, tachycardia, apnea, feeding problems, irritability, and/or apathy. Late-onset sepsis was defined as a positive blood culture after 72 h of life [11]. If the blood culture did not turn positive but clinical signs implied antibiotic treatment for 7 days, late-onset sepsis was considered probable but not proven. If in one of the episodes a causal microorganism was found, the infant was classified as proven infection.

Clinical data were collected from the patients’ medical charts. Patient variables included gestational age at birth, birth weight, gender, days of ventilation, > 24 h steroid treatment for either respiratory or circulatory insufficiency, intraventricular hemorrhage (IVH) and periventricular leukomalacia (PVL) according to Volpe [12], lumbar puncture, hemodynamic significant patent ductus arteriosus (HS-PDA), surgery, necrotizing enterocolitis (NEC) stage 2 or 3 according to Bell [13], and bronchopulmonary dysplasia (BPD) according to Jobe [14]. Surgical interventions were defined as abdominal, cardiac, and ophthalmological surgery or surgery for other indications. Positive blood cultures were divided in two groups: coagulase-negative staphylococci and other microorganisms for subanalyses.

All surviving patients were invited for outpatient clinic follow-up at term age and at 3, 6, 12, and 24 months corrected age. During this visit, physical examination was performed, including biometrics. At 2 years corrected age, neurodevelopment was assessed according to Bayley’s Scales of Infant and Toddler Development II (BSID-II) [15, 16], lexiquotient [17, 18], and Child Behavior Checklist (CBCL) [19] by trained pediatric psychologists, who were aware of participants to the study, though unaware of in which study group the patients belonged. Maternal education level was registered.

Statistical analysis was performed with SPSS using version 22 (SPSS Inc., Chicago, IL, USA). Statistical analyses were performed with chi-square using Fisher’s exact test as appropriate for dichotomous data and *T* test for paired data. For analysis of two or more groups and confounding, multivariate linear regression techniques were used. Possible confounders were need for invasive ventilation, need for inotropic agents, postnatal corticosteroids, PVL, NEC, or BPD. Confounding was defined as change in regression coefficient over 10%. A probability *p* value < 0.05 was considered statistically significant.

## Results

**Fig. 1:**
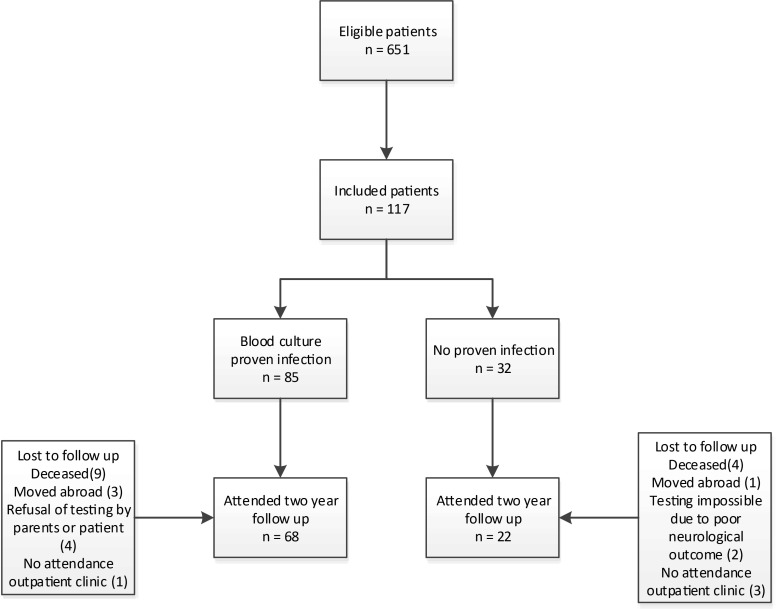
Patient inclusion

Patient enrollment and inclusion are demonstrated in Fig. 1 and patient characteristics are described in Table 1. During the inclusion period, 651 patients would have been eligible for inclusion, of which 117 patients were included during an episode of suspicion of infection. Ninety-one patients did not experience an episode of suspicion of infection and therefore were not included in the study. Of the 117 patients with clinical suspicion of late-onset sepsis, 19 patients had 2 episodes (of which in 6 patients, both blood cultures turned positive, in 10 patients, 1 blood culture turned positive, and in 3, none of the blood cultures turned positive), and 1 had 3 episodes (of which one with positive blood culture) of suspicion of late-onset sepsis.

**Table 1:** Patient characteristics for suspected late-onset sepsis (*n* = 117)

	Total (*n* = 117)	Proven LOS (*n* = 85)	No proven LOS (*n* = 32)	*p* value
Gestational age (wks, SD in days)	28 1/7 (16 days)	28 0/7(15 days)	28 2/7 (19 days)	0.713
Gestational age < 27 0/7 weeks (*n*)	33	24	9	> 0.999
Gestational age 27 0/7–28 5/7 weeks (*n*)	44	31	13	0.675
Gestational age ≥ 29 0/7 weeks (*n*)	40	30	10	0.827
Birth weight (g)	1061 (330)	1078 (322)	1016 (350)	0.364
Birth weight SDS	0.08 (1.21)	0.16 (1.02)	− 0.14 (1.61)	0.330
SGA (SDS < 2 SDS)	5	2	3	0.125
Apgar score 5 min	7.4 (1.75)	7.3 (1.9)	7.7 (1.4)	0.190
Umbilical cord pH, arterial	7.27 (0.11)	7.27 (0.11)	7.27 (1.37)	0.936
Male	49.6%	50.6%	46.9%	0.863
Survival	89.7%	89.4%	87.5%	0.259

Mean (*SD* standard deviation), *LOS* late-onset sepsis, *wks* weeks, *SGA* small for gestational age, *SDS* standard deviation score, *T* test and Fisher’s exact test

In Table 2, the causal agents in the blood culture are described. If patients had more than one infectious, episode the causal agents were stated separately. In 32 of the 117 patients, blood culture revealed no microorganisms; three of these patients had two clinical infectious episodes in which both blood cultures were negative. Of the 117 patients suspected of late-onset sepsis, 85 (73%) were proven with positive blood culture. Of the 32 patients with negative blood cultures, 14 patients (12%) were treated with antibiotics for 7 days due to persistent clinical symptoms and in 18 patients (15%), antibiotics were discontinued after 48 h due to negative blood culture and recovered of clinical symptoms for suspicion of late-onset sepsis.

**Table 2:** Causal infectious agents in blood culture

Causal microorganism	Incidence (number of cultures)
Coagulase-negative staphylococci	70
*Staphylococcus aureus*	14
*Escherichia coli*	2
*Klebsiella oxytoca*	2
*Enterococcus faecalis*	1
*Streptococcus agalactiae*	1
Gram-positive rod, not further specified	1

In 68 patients, cerebral fluid could be obtained for culture. In 61 patients, culture of cerebral fluid showed no microorganisms. In three coagulase-negative staphylococci and in four patients, *Staphylococcus aureus* were isolated. No significant differences could be found in neurodevelopment between patients with proven meningitis and those with no meningitis.

In Table 3, significant comorbidity is stated. No significant differences were found in comorbidity between groups with or without proven infection.

**Table 3:** Major comorbidity

Morbidity	Total incidence n (%)	Proven LOS (*n* = 85)	No proven LOS (*n* = 32)	*p* value
RDS ≥ grade III	37 (31.7)	24 (28.2)	13 (40.6)	0.265
Need for mechanical ventilation	59 (50.4)	42 (49.4)	17 (53.1)	0.836
NEC ≥ grade II	20 (17.1)	16 (18.8)	4 (12.5)	0.584
Need for inotropes	3 (2.6)	3 (3.5)	0 (0)	0.561
IVH ≥ grade II	13 (11.1)	9 (10.6)	4 (12.5)	0.749
PVL	6 (5.1)	4 (4.7)	2 (6.3)	0.664
Surgical intervention	9 (7.7)	7 (8.2)	2 (6.3)	> 0.999
Corticosteroid use postpartum	19 (16.4)	14 (16.5)	5 (15.6)	> 0.999
BPD	23 (19.7)	14 (16.5)	9 (28.1)	0.193

*LOS* late-onset sepsis, *RDS* respiratory distress syndrome, *NEC* necrotizing enterocolitis, *IVH* intraventricular hemorrhage, *PVL* periventricular leukomalacia, *BPD* bronchopulmonary dysplasia, Fisher’s exact test

Table 4 shows the results of the BSID-II, lexiquotient, and Child Behavior Checklist at 2 years corrected age. Of the cohort of 117 patients, 90 patients were included in the analyses for long-term follow-up, of which 68 patients in the proven and 22 patients in the non-proven infection group. Reasons for lost to follow-up were deceased (13, of which 12 during NICU admission and one after NICU discharge), moved abroad (4), refusal for neurodevelopmental testing by parents or patient (4), impossible testing due to poor neurodevelopmental outcome (1 due to cerebral palsy and 1 due to a cortical migration disorder), and no attendance to outpatient clinic (4).

**Table 4:** Comparison of BSID-II, Lexilijst, and child behavioral scores at 2 years of corrected age

	Proven LOS (*n* = 68)	No proven LOS (*n* = 22)	*p* value
Corrected age at testing	24 m 15 d (47 d)	24 m 12 d (28 d)	0.759
Composite cognitive score (BSID-II)	100 (9.0)	98 (13.90)	0.276
Composite motor score (BSID-II)	100 (9.4)	99 (12.3)	0.687
Lexiquotient (Lexilijst)	91 (16.1)	88 (18.2)	0.489
Total behavioral score (CBCL)	26 (14.9)	30 (21.2)	0.283
Total internalizing score (CBCL)	5 (4.3)	8 (7.9)	0.171
Total externalizing score (CBCL)	12 (7.5)	12 (7.6)	0.908

Mean (standard deviation). *LOS* late-onset sepsis, *m* months, *d* days. *T* test

In the proven late-onset sepsis group, 68 patients were tested (80%); in the non-proven late-onset sepsis group, 22 (69%). No statistical significant differences were found in cognitive, motor, and behavioral scores or in lexiquotient, indicating comparable neurodevelopmental outcomes in both groups. Also, when comparing the proven late-onset sepsis group (68 patients tested, 80%) with the group in which antibiotics were discontinued after 48 h (12 patients tested, 38%), no significant differences could be demonstrated in either of these neurodevelopmental outcome scores.

In Table 5, the effects of infection on the different domains are presented. Confounding analyses revealed PVL and BPD as confounding factors. When correcting for those confounders (PVL and BPD), the effects decrease substantially, except the effect on behavioral score.

**Table 5:** Effects on neurodevelopment no proven versus proven late-onset sepsis

	Regression coefficient	95% confidence interval	*p* value
Composite cognitive score, crude	2.567	− 2.090 – + 7.223	0.276
Composite cognitive score, adjusted*	1.892	− 2.871 – + 6.655	0.432
Composite motor score, crude	1.022	− 4.003 – + 6.047	0.687
Composite motor score, adjusted*	0.746	− 4.452 – + 5.943	0.776
Lexiquotient, crude	3.520	− 6.605 – + 13.644	0.489
Lexiquotient, adjusted*	0.871	− 9.038 – + 10.780	0.861
Total behavioral score, crude	− 4.776	− 13.564 – + 4.012	0.283
Total behavioral score, adjusted*	− 5.139	− 16.019 – + 3.672	0.241

*Adjusted for confounding by bronchopulmonary dysplasia and periventricular leukomalacia. Linear regression

Maternal highest educational level was also taken into account. No differences in maternal education between the groups were found (*p* = 0.381). In multivariate analyses, no significant difference could be shown of maternal education.

The group experiencing late-onset sepsis had a trend to a higher risk on poor outcome, defined as BSID-II composite cognitive and/or motor score ≤ 1 SD of general population scores, or dead (*p* = 0.068).

Early markers of possible brain damage, e.g., echo densities on cerebral ultrasound, were not associated with differences in the different domains of neurodevelopment (cognitive, motor, and behavioral scores or lexiquotient). Also, when comparing the major ultrasound abnormalities, no significant differences could be demonstrated.

**Fig. 2:**
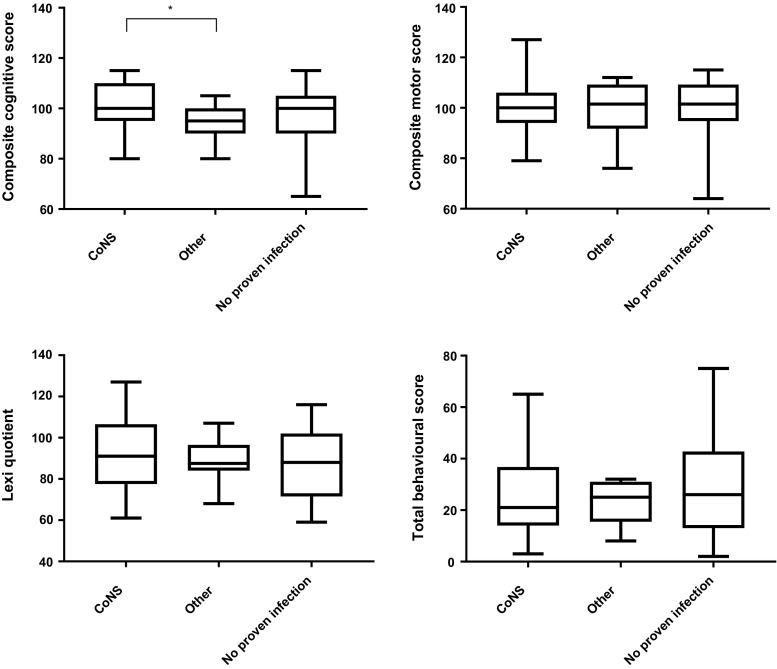
Comparison of BSID-II, Lexilijst, and child behavioral scores at 2 years of age of CoNS, other infections, and no proven infection groups (*p = 0.007; *T* test, *CoNS* coagulase-negative staphylococci)

Due to the high representation of coagulase-negative staphylococci (55 patients of the follow group), with a possible milder clinical course and long-term effects, the study population has been divided in three groups: coagulase-negative staphylococci, other, and no proven infection. A significant difference was found in the composite cognitive score subscale (*p* = 0.016). Subanalyses show the difference in favor of the coagulase-negative staphylococci group versus the other causal infectious agent group (*p* = 0.007) (see Fig. 2). No significant differences were found in the other subscales (composite motor score, lexiquotient, and behavioral scores) between the three groups.

## Discussion

Long-term neurodevelopmental outcomes after admission at the NICU for prematurity are at risk. One of the potential risk factors is late-onset sepsis. Therefore, the present study investigated the effects on long-term psychomotor outcome in preterm infants suspected of late-onset sepsis.

No differences in neurodevelopmental outcome at 2 years corrected age could be demonstrated between groups with or without proven infection. One could suggest this last group might have been going through serious other comorbidities, e.g., intracerebral pathology, protracted respiratory support for which postnatal corticosteroids were administered, and therefore demonstrate impaired neurodevelopmental outcome. However, these differences in comorbidity in both subgroups could not be demonstrated as shown in Table 3.

Coagulase-negative staphylococci mono-infection represented more than half of all episodes of late-onset sepsis, in 65 patients (55%). The other four had a second episode with a positive blood culture with another microorganism. In literature, high incidences of coagulase-negative staphylococci are reported [3]. Conflicting results of coagulase-negative staphylococci infections have been reported in previous studies. Alshaikh et al. state coagulase-negative staphylococci are associated with an increased risk for cognitive delay and major disability [20]. In contradiction, Mittendorf et al. state these infections are not associated with poor neurodevelopmental outcome [21]. Previous studies in infants with coagulase-negative staphylococci infections showed a low C-reactive protein (CRP) in approximately one-third of the episodes [22]. Possibly, the inflammatory cascade is less activated. This might suggest a lower inflammatory response and possibly less detrimental effects on long-term neurodevelopmental outcome.

In our study, we demonstrated a significant difference for composite cognitive score in favor of patients experiencing late-onset sepsis caused by coagulase-negative staphylococci in comparison to other causative agents. However, it is important to note that the majority of this cohort attending follow-up experienced coagulase-negative staphylococci sepsis (55/90). Thirteen patients attending follow-up had a causative agent other than coagulase-negative staphylococci. No differences were demonstrated in the other domains. Long-term cognitive outcome is also known to be influenced by maternal education [23]. However, no significant effect of maternal education level could be demonstrated. In future studies, the influence of these socioeconomic effects should be studied more extensively.

In an earlier publication, cerebral ultrasound abnormalities were explored as an early indicator for adverse neurodevelopmental outcome. In line with the relatively good prognosis of coagulase-negative staphylococci infections, we did not find major brain damage in this cohort using cerebral ultrasound [24]. This finding is in consistency with the normal scores on the BSID-II at 2 years of age, corrected for prematurity.

The present study has some limitations. Due to the high representation of coagulase-negative staphylococci, the effects of other infectious agents are underexposed. On the other hand, the high incidence of coagulase-negative staphylococci is a common practice in level III NICUs and thus representative for this patient group.

The control group consisting of patients without proven infection is relatively small in comparison with the proven infection group. In addition, the group without proven infection can contain patients with an infection, but not have a positive blood culture. This might influence the neurodevelopmental outcome negatively in the control group, and therefore, differences between groups become less. However, no statistical different neurodevelopmental outcome could be demonstrated in subanalysis comparing the proven infection group with the group in which antibiotics were discontinued after 48 h. One could also argue the load of infectious agents is lower, and possibly, the inflammatory cascade is less activated as mentioned before. Future research into inflammatory cytokines and their role in the inflammatory cascade and subsequent effect on neurodevelopmental outcome might give further insights. Also, a larger cohort with a substantial control group, and considerable long-term follow-up [25], might elucidate possible effects of inflammation in patients suspected of late-onset sepsis with negative blood culture. Though a control group with preterm infants without any sign of infection will be challenging. Surrogate markers as CRP or procalcitonin might be needed to construct a “control group” [26].

## Conclusion

In this study, in preterm-born infants, no differences in neurodevelopmental outcome at 2 years corrected age could be demonstrated between the groups with or without proven infection on neurodevelopmental outcome at 2 years corrected age in preterm infants. This finding needs replication in larger cohorts with a substantial control group. Patients experiencing coagulase-negative staphylococci sepsis have a significantly better cognitive outcome in comparison to other causal infectious agents.

## Abbreviations

BPD: Bronchopulmonary dysplasia
BSID: Bayley’s Scale of Infant and Toddler Development
CBCL: Child Behavior Checklist
CP: Cerebral palsy
CRP: C-reactive protein
HS-PDA: Hemodynamic significant patent ductus arteriosus
IVH: Intraventricular hemorrhage
NEC: Necrotizing enterocolitis
NICU: Neonatal intensive care unit
PVL: Periventricular leukomalacia
RDS: Respiratory distress syndrome
